# Lévy Flights Diffusion with Drift in Heterogeneous Membranes

**DOI:** 10.3390/membranes13040417

**Published:** 2023-04-07

**Authors:** Anna Strzelewicz, Monika Krasowska, Michał Cieśla

**Affiliations:** 1 Faculty of Chemistry, Silesian University of Technology, Strzody 9, 44-100 Gliwice, Poland; monika.krasowska@polsl.pl; 2 Institute of Theoretical Physics, Jagiellonian University, Łojasiewicza 11, 30-348 Kraków, Poland; michal.ciesla@uj.edu.pl

**Keywords:** diffusion, drift, heterogeneous membrane, structure, simulation, Lévy flights

## Abstract

The modelling of diffusion in membranes is essential to understanding transport processes through membranes, especially when it comes to improving process efficiency. The purpose of this study is to understand the relationship between membrane structures, external forces, and the characteristic features of diffusive transport. We investigate Cauchy flight diffusion with drift in heterogeneous membrane-like structures. The study focuses on numerical simulation of particle movement across different membrane structures with differently spaced obstacles. Four studied structures are similar to real polymeric membranes filled with inorganic powder, while the next three structures are designed to show which distribution of obstacles can cause changes in transport. The movement of particles driven by Cauchy flights is compared to a Gaussian random walk both with and without additional drift action. We show that effective diffusion in membranes with an external drift depends on the type of the internal mechanism that causes the movement of particles as well as on the properties of the environment. In general, when movement steps are provided by the long-tailed Cauchy distribution and the drift is sufficiently strong, superdiffusion is observed. On the other hand, strong drift can effectively stop Gaussian diffusion.

## 1. Introduction

Simulations of diffusion in membranes are fundamental for understanding transport processes through membranes, especially when it comes to improving process performance. The diffusion type is determined by the microscopic dynamics and properties of an environment, such as the polymer structure and related internal changes resulting from the permeation of mixture components [1,2,3,4]. Typically, a particle passing through a medium constantly interacts with other particles and other components of the system. These collisions result in irregular observable movements, called Brownian motion. An example of an equation describing the dynamics of a single particle at the microscopic level is the Langevin equation. It is a stochastic differential equation, and its solutions, i.e., the trajectories corresponding to the same initial conditions, differ. Numerous collisions of the studied particle with other particles and the medium can be approximated by a random force, the properties of which depend on the chosen assumptions [5,6,7]. In the idealized model of such motion, the random force **R**(t) acting on a particle corresponds to a Gaussian process. This means that the collisions are independent and are described by a probability distribution with moments that are well defined [8,9]. This assumption leads to so-called normal diffusion; as a consequence of the assumption of collision independence, it has a Markovian character and linear scaling of the mean square displacement with time:

(1) $$\langle \Delta r^{2}\rangle =2nDt$$

This relation is called the Einstein relation, where *D* is the diffusion coefficient, *n* is the spacial dimension (n=1,2,3), *t* is time, and $\langle \Delta r^{2}\rangle$ is the mean square displacement (MSD) [10,11].

The above relation can be generalized to other processes where the mean square displacement depends on time, as follows:

(2) $$\langle \Delta r^{2}\rangle ∼t^{\alpha }$$

**Figure 1:**
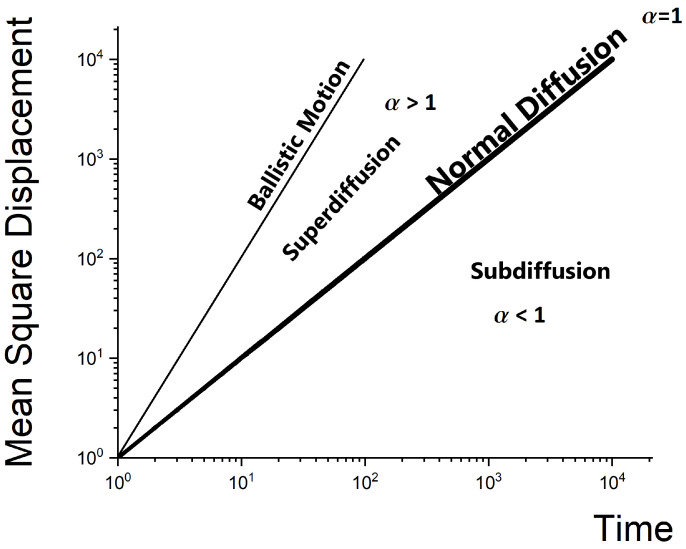
Anomalous diffusion regimes as characterized by Equation (2), i.e., power-law scaling of the mean squared displacement (MSD) with time.

In general, when α=1 we have normal diffusion (see Equation (1) and Figure 1). When α<1, the particle covers a smaller area than in normal diffusion at the same time. This movement is called subdiffusion. When α>1, the particle covers a larger area; such a movement is called superdiffusion. We call both cases anomalous diffusion [10,12]. Anomalous diffusion behavior (see Equation (2)) is closely related to the breakdown of the central limit theorem, and is caused by either broad distributions or long-range correlations. Alternatively, anomalous diffusion relies on the validity of the Lévy–Gnedenko generalized central limit theorem for situations in which not all moments of the underlying elementary transport events exist. Thus, wide spatial jumps or waiting time distributions lead to non-Gaussian propagators, and possibly non-Markovian time evolution of the system [13,14].

Subdiffusion is usually caused by the trapping of a randomly moving object, while superdiffusion is the consequence of unlimited variance in the distance between successive object positions [9,15]. However, the following point remains worth considering. Using a continuous-time random walk (CTRW) description [16], it can be shown that if the jump length and the times between jumps are distributed via the power-law asymptotics $p\left(r\right)\propto {\left|r\right|}^{-1-\mu },p\left(t\right)\propto t^{-1-\nu }$ (with 0<μ<2, 0<ν<1), and $p\left(r,t\right)=t^{-\nu /\mu }p\left(rt^{-\nu /\mu },1\right)$, then the second moment of p(r,t) and $t^{2\nu /\mu }$ scale similarly. It may happen that 2ν=μ, in which case the ensemble average leads to a linear signature of time-dependent MSD even for jump lengths from heavy-tailed distribution. This is called paradoxical diffusion [15,17,18].

Lévy walks [19,20] and Lévy flights [21] are anomalous diffusive forms of random motion that are widely observed in natural systems, including movements of animals [22]. They are characterized by a grouping of short steps that are occasionally interspersed with longer movements; as such, they represent superdiffusive processes and can travel much further from the starting position than a Brownian walk of the same duration. The main difference between Lévy walks and Lévy flights is that the former has a finite propagation velocity and continuous trajectory, which makes it inertial and easier to apply to physical systems. From the numerical perspective, in both these types of movements the jump lengths are selected from the same distribution, while for Lévy walk a constant velocity is assumed, which means that the time of a single movement varies. In Lévy flights, the jump is applied instantaneously. It should be noted that Levy flights can be used as an approximation of Lévy walks [23].

In this study, we focus on one specific type of Lévy flight in which jump lengths are drawn independently from the Cauchy distribution:

(3) $$p_{C}\left(x\right)=\frac{\sigma^{2}}{\pi \left(\sigma^{2}+x^{2}\right)},$$

where σ denotes the width of the distribution. Because the Cauchy distribution is α-stable, the tracer displacement after *t* independent jumps is the Cauchy distributed random variable, though now of a width σt [24]. Considering that we are interested in square displacement, let $y\left(t\right)=x^{2}\left(t\right)$. Thus, the probability distribution function of y(t) is

(4) $$p\left[y\right]=\frac{\sigma t}{\pi \sqrt{y}\left(\sigma^{2}t^{2}+y\right)}$$

However, the mean square displacement for the Cauchy distributed random variable is ill-defined; therefore, the median is used instead. In this case, the median of *y* is provided by the following equation:

(5) $$\frac{1}{2}=\int_{0}^{M[y]}p\left[y^{\prime }\right]dy^{\prime }=\frac{2\tan^{-1}\left(\frac{\sqrt{M[y]}}{\sigma t}\right)}{\pi },$$

which leads directly to

(6) $$M\left[y\right]=\sigma^{2}t^{2}.$$

**Figure 2:**
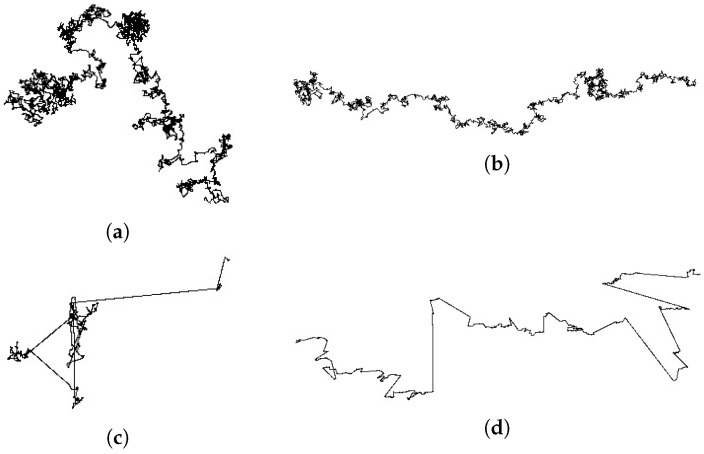
Comparison of Gaussian (Brownian) and Lévy flight in open space. The top panels show diffusion caused by the random Gaussian distribution of steps and the bottom ones show Cauchy distributed steps. In the left panels, there is no external drift, while in the right panels there is a drift along the horizontal axis. (**a**) The trajectory of Brownian motion without drift, (**b**) trajectory of Brownian motion with drift, (**c**) trajectory of Lévy flight without drift, and (**d**) trajectory of Lévy flight with drift.

Figure 2 shows example trajectories of particle motion in four cases: Gaussian (Brownian) motion with and without drift and Lévy flight with and without drift. A motion analysis of a single particle is a standard tool to probe the local physical properties of complex systems because it delivers the complete, or at least projected, trajectory of an individual particle. In open space, the particle moves around its initial position and slowly away from it, with no preference for the direction of movement (Figure 2a). The addition of drift causes a distinct movement of the particle in the direction of the drift (Figure 2b). In the case of Lévy flights, the particle moves around a certain point while alternating with a ’long jump’ along a straight line (Figure 2c). Introducing drift into this type of motion results in more jumps having a preferred direction (Figure 2d).

The main purpose of this study is to check how the above general picture of diffusion applies to transport properties in heterogenic membranes. In our previous studies [25,26], we modelled structures of heterogenic membranes which resemble real membrane structures, i.e., sodium alginate membranes filled with iron oxide nanoparticles [26]. Subdiffusive motion is observed in a crowded environment regardless of whether particle movement is induced by a Gaussian or Lévy flight process [24,27]. On the other hand, the existence of an external force that causes constant drift can speed up the transport of Brownian particles through a crowded environment [26,28], while when the amount of the drift is too high the transport is practically stopped. In this study, we want to test the last possible situation, i.e., when the external drift is combined with Lévy flights. Because we compare the obtained results with previous studies on transport in heterogenic membranes, we use numerically modelled structures that resemble them [29].

The rest of this paper proceeds as follows: Materials and Methods, Results, Discussion, and Conclusions. Section 2 (Materials and Methods) discusses the membranes and the methodology used to study Lévy flight diffusion on these membranes with and without drift. Section 3 (Results and Discussion) provides a detailed description of the study results and graphs, analyzes the results, and provides an interpretation. Finally, the manuscript closes with our Conclusions (Section 4).

## 2. Materials and Methods

### 2.1. The Model Membranes

**Figure 3:**
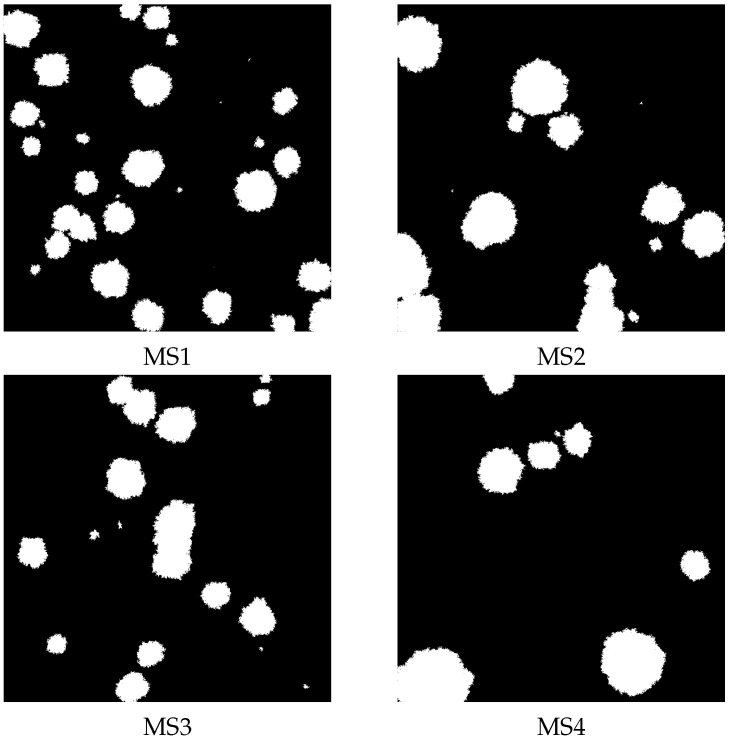
The artificial membrane structures with different structural parameters: MS1 (ρ = 0.85, $d_{f}$ = 1.9687, ΔD = 0.7102); MS2 (ρ = 0.85, $d_{f}$ = 1.9664, ΔD = 1.1809); MS3 (ρ = 0.9, $d_{f}$ = 1.9792, ΔD = 0.8084); and M4 (ρ = 0.9, $d_{f}$ = 1.9783, ΔD = 1.6377).

We generated images of the structure of artificial membranes using the method described in [25,29]. Similarly, we generated four stuctures, named MS1, MS2, MS3, and MS4 (see Figure 3), with the following parameters: MS1 (ρ = 0.85, $d_{f}$ = 1.9693, ΔD = 0.4259); MS2 (ρ = 0.85, $d_{f}$ = 1.9664, ΔD = 1.1809); MS3 (ρ = 0.9, $d_{f}$ = 1.9797, ΔD = 0.3667); and M4 (ρ = 0.9, $d_{f}$ = 1.9783, ΔD = 1.6377).

**Figure 4:**
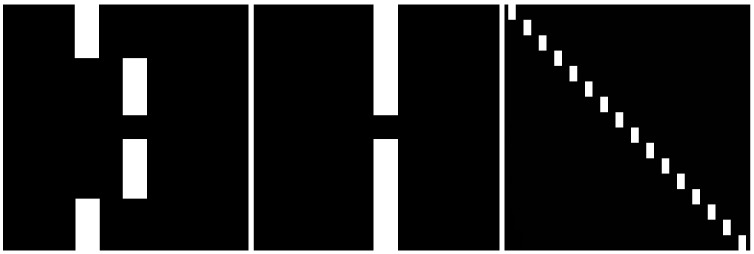
Artificial membrane structures with obstacles in different positions. These three structures are designed to show which distributions of obstacles can cause changes in transport. **Left panel:** The first structure has three breaks (structure S1). **Central panel:** The second structure has only one break, at the central point of the membrane (structure S2). **Right panel:** The third structure has obstacles aligned along the main diagonal of the structure (structure S3). The breaks between the obstacles are equally distributed.

When analyzing Lévy flight diffusion with drift on structures MS1–MS4 where obstacles are randomly distributed, we decided to prepare structures with deterministically distributed obstacles. We prepared three artificial membrane structures with differently positioned obstacles in order to show which distributions of obstacles can cause changes in transport. The first structure has three breaks (structure S1 in Figure 4 (left panel)). The second structure has only one break, at the central point of the membrane (structure S2 in Figure 4 (central panel)). The third structure has obstacles aligned along the main diagonal of the structure (structure S3 in Figure 4 (right panel)). The breaks between the obstacles are equally distributed.

The obstacle positioning we chose allowed us to determine how the placement of obstacles affects the movement of particles and whether effective diffusion in membranes with and without external drift depends on the type of internal mechanism that causes the movement of particles.

### 2.2. Simulations of Gauss and Cauchy Flights

We used numerical simulations to analyze the diffusion in the membrane systems mentioned above. A set of 100 randomly distributed independent tracers penetrated the available space, i.e., the black regions of the membranes shown in Figure 3 and Figure 4, according to the conditions presented below.

A single movement of a tracer is

(7) $${\vec{x}}_{i}=\left[x_{i}\cos\phi ,\;x_{i}\sin\phi \right],$$

where $|x_{i}|$ denotes the length of the step and ϕ is its direction.

The length of the step $x_{i}$ is randomly selected from Gaussian

(8) $$p_{G}\left(x\right)=\frac{1}{\sqrt{2\pi }\sigma }\exp\left[-\frac{x^{2}}{2\sigma^{2}}\right]$$

or Cauchy (3) distributions with parameter σ=1. The direction ϕ is selected according to the uniform distribution on the interval [0,2π). To generate normally distributed random variables, we used the Box–Muller transform [30]. The Cauchy distribution can be obtained from the uniform one using $\tan\left[\pi \left(u-1/2\right)\right]$, where *u* is the uniformly distributed random number on the interval [0,1). Having determined the step ${\vec{x}}_{t}$, we next checked whether or not a line between the present position $\vec{r}\left(t\right)$ and the target position $\vec{r}\left(t\right)+{\vec{x}}_{t+1}$ crossed any obstacle or ended on it, and whether the tracer changed its position ($\vec{r}\left(t+1\right)=\vec{r}\left(t\right)+{\vec{x}}_{t}$). Otherwise, it was assumed that the tracer was reflected and returned to its previous position ($\vec{r}\left(t+1\right)=\vec{r}\left(t\right)$). The simulation was stopped after $10^{7}$ such iterations. During the numerical calculations, the set of 100 independent tracer trajectories was recorded. The time was measured using the number of iterations and the distance; if not explicitly stated otherwise, it was measured in pixels. To avoid saturation of square displacement due to the finite size of the membrane, periodic boundary conditions were used. The dependence of the median time for Cauchy flights and the mean value of the square displacement $x^{2}$ of the time for Gaussian processes determined the effective diffusion exponent α defined in Equation (2).

Additionally, the diffusion was analyzed in terms of the effective exponent α (see (2)); however, for Cauchy flights we used the median instead of the mean value:

(9) $$M\left[x^{2}\right]\left(t\right)∼\langle x^{2}\rangle ∼t^{\alpha }\;\;\mathrm{for}\mathrm{large}t.$$

The effective diffusion exponents were estimated by fitting the power law (9) to the MSD data for $t>10^{5}$. Thus, the effective diffusion exponent at long time limits was a slope of the line fitted to the data in the double logarithmic plot:

(10) $$\log\langle x^{2}\rangle =\alpha \log t.$$

## 3. Results and Discussion

**Figure 5:**
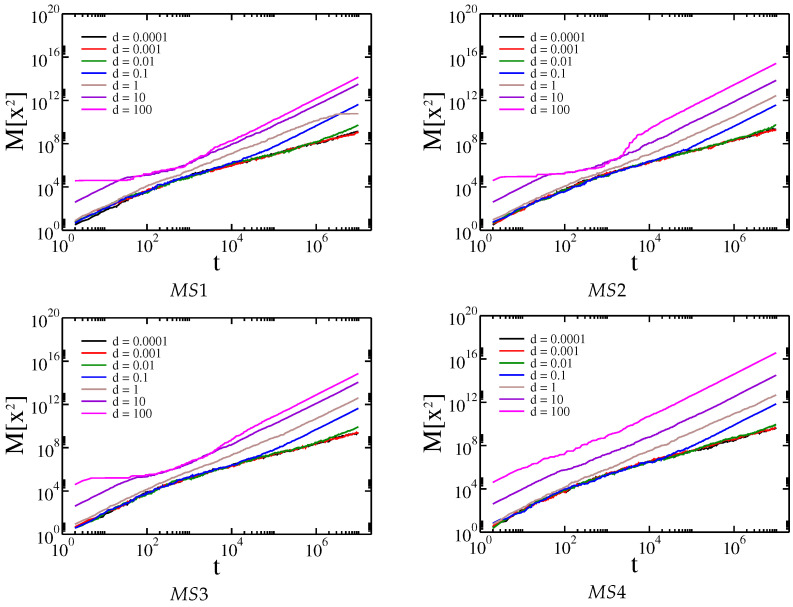
Dependence of the median of the square displacement $x^{2}$ on time driven by Cauchy flights moving in the structures MS1, MS2, MS3, and MS4, respectively.

In the previous section, we have described the basis of Gaussian and Cauchy flight simulations. Simulations of Cauchy flights with and without drift were performed first for the artificial membrane structures MS1, MS2, MS3, and MS4 presented in Figure 3. These artificial structures resemble the real membrane morphology of sodium alginate membranes filled with iron oxide nanoparticles. The distribution of obstacles in the membrane is random. For real membranes, the distribution of powder depends on the method of membrane preparation and the type of powder used. The type of powder affects the random distribution of powder and aggregate formation. The generated structures correspond to real structures; consequently, the distribution of obstacles is random. The dependence of the median of the square displacement $x^{2}$ on time driven by Cauchy flights moving in the structures MS1, MS2, MS3, and MS4 is shown in Figure 5.

**Figure 6:**
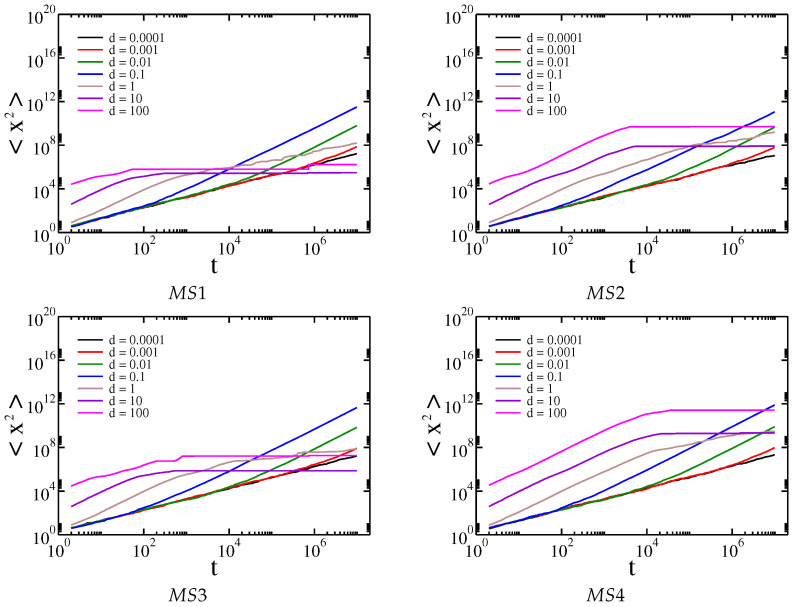
Dependence of the mean square displacement $x^{2}$ on time driven by Gaussian random walk in the structures MS1, MS2, MS3, and MS4, respectively.

For comparison, the mean square displacement for Gaussian random walk on the same membranes is shown in Figure 6.

**Figure 7:**
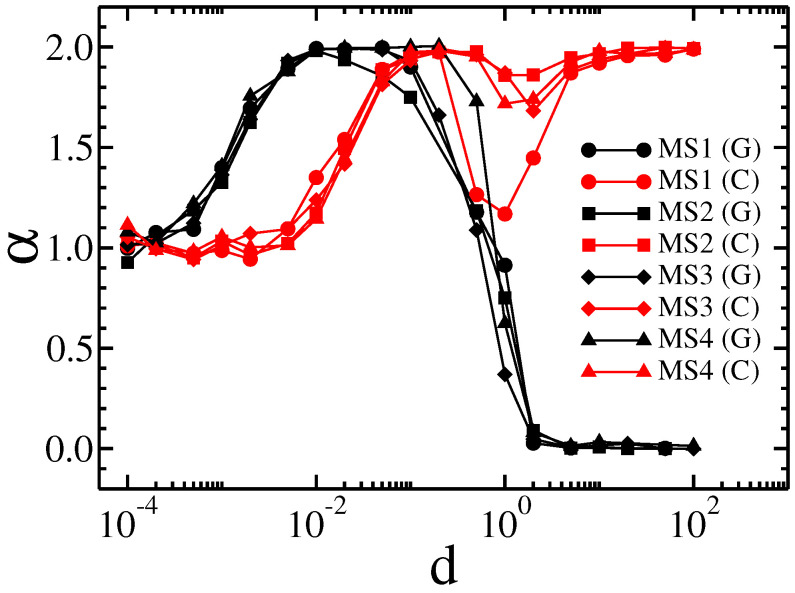
The dependence of the effective diffusion exponent α on the drift *d* for Gaussian random walks and Lévy flights on membranes MS1, MS2, MS3, and MS4.

For each membrane, a similar pattern is observed. In the beginning, the median of square displacement for Cauchy flights grows superlinearly. Then, for relatively small drift, the movement slows down, which confirms the previous results that the effective diffusion between obstacle types is generally determined by the properties of the environment and not by the internal process that causes the movement of the tracers [24,25,26,27,28,29,31]. For medium and high drifts, the movement is asymptotically superlinear; however, in the case of high drift, we observe a slowing down at medium times. This is because when a tracer hits an obstacle it requires time to avoid it. This time is longer when the drift is higher, because the tracer is pushed harder into the obstacle and the probability of drawing a step large enough to cancel the drift is relatively small. It is worth noting that for large drifts and Gaussian random walks the movement is entirely stopped, as the probability of drawing a large number decreases exponentially with its value. The above observations can be supported by the analysis of the effective diffusion exponent α, which is shown in Figure 7.

Here, we notice that the effective exponent α for Gaussian random walks begins to grow with a smaller amount of drift than is the case for Cauchy flights. Additionally, for a larger drift that stops Gaussian diffusion, the local minimum of α is present for Cauchy flights.

**Figure 8:**
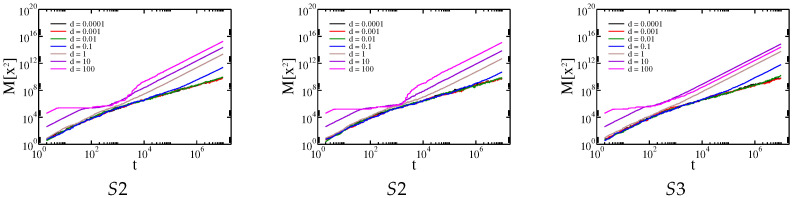
Dependence of the median of the square displacement $x^{2}$ on the time driven by Cauchy flights moving in the structures S1, S2, and S3, respectively.

**Figure 9:**
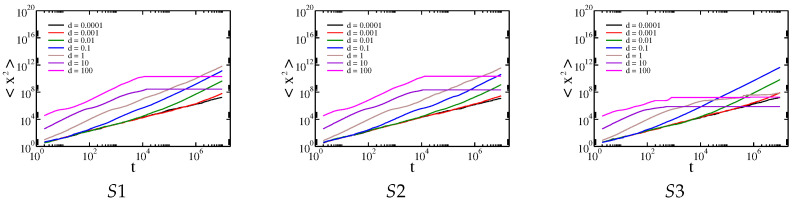
Dependence of the average of the square displacement $x^{2}$ on time driven by Gaussian random walk in the structures S1, S2, and S3, respectively.

In addition to the qualitatively same results for all membranes studied, there are a number of quantitative differences. For example, for Cauchy flights diffusion slowdown is not observed for the MS4 membrane, whereas it is present for the others. Therefore, we decided to generate specific sample structures of obstacles to determine the key factor in the type of observed results. Artificial membrane structures with differently placed obstacles are presented in Figure 4. The first structure has three breaks (structure S1). The second structure has only one break, at the central point of the membrane (structure S2). The third structure has obstacles aligned along the main diagonal of the structure (structure S3). Gaussian and Cauchy flight simulations with and without drift were performed on all these structures. The dependence of the median square displacement for Cauchy flight is shown in Figure 8, and the mean square displacement for the Brownian motion is shown in Figure 9.

**Figure 10:**
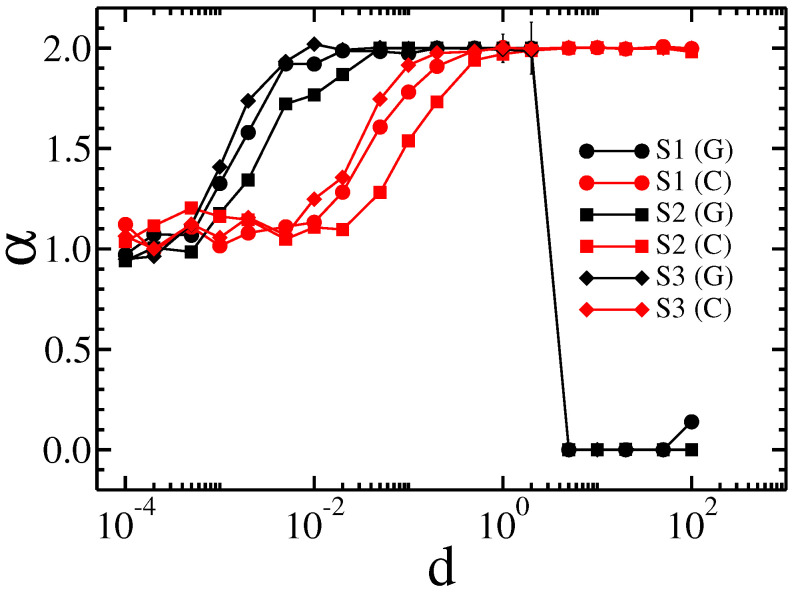
The dependence of the effective diffusion exponent α on the drift *d* for Gaussian random walks and Lévy flights in the membranes S1, S2, and S3.

The effective diffusion exponent for both types of transport is shown in Figure 10.

In general, all the previously observed effects are present here. However, we can conclude that larger and more solid obstacles cause diffusion slowdown for intermediate times and Cauchy flights (see the results for structures S1 and S2 in comparison with those for S3). Because obstacles block most of the area and there are only small spaces in which to avoid them, the Gaussian random walk is efficiently timed by a sufficiently large drift. On the other hand, a small amount of drift d≪σ mainly speeds up Gaussian diffusion. This is due to the character of the Gaussian and Cauchy distributions. In the first case, most of the steps are comparable to σ. For the Cauchy distribution, there is generally a smaller step size, while from time to time a large leap occurs. However, these leaps are stopped by obstacles, while the small steps are not as efficient as in the case of the Gaussian distribution.

When d≫σ and the tracer hits an obstacle, it requires a relatively large step size to cancel the effect of the drift, which is quite probable for the Cauchy distribution and almost impossible for the Gaussian distribution. Therefore, a rapid decrease in effective transport is observed for Gaussian diffusion, while the movement induced by Cauchy flight is barely affected.

## 4. Conclusions

The present study was designed in order to understand the relationship between membrane structures and their diffusive transport characteristics in the presence of external drift. The study focused on numerical simulation of particle movement in different membrane structures with differently spaced obstacles. Four of the studied structures were similar to the structures of sodium alginate membranes filled with iron oxide nanoparticles. The other three structures were designed specially to show the crucial factors that affect most diffusional transport under the influence of drift. We investigated the movement of the particles as driven by Gaussian random walks and Cauchy flights, showing that effective diffusion in membranes with external drift depends on the amount of drift, the type of internal mechanism that causes the movement of the particles, and the distribution of the obstacles. In cases of weak drift, the effective diffusion is fully determined by the environment (i.e., the properties of the membranes), whereas the internal mechanism (i.e., Cauchy flight or Brownian motion) does not matter. For higher drift, superdiffusion is recognized; however, when the drift is too strong, Brownian motion is almost stopped, as the tracers are constantly pushed against the obstacles. Due to the heavy-tailed distribution of Cauchy flights, this pushing can be overcome by random motion; thus, transport can continue with the effective exponent α≈2. In general, the observed relations do not depend qualitatively on the morphology of the studied structures.

## Data Availability

The numerical data, analyzed images, and necessary software are available on reasonable request.

## Abbreviations

The following abbreviations are used in this manuscript:

α	Effective diffusion exponent
*d*	Drift
$d_{f}$	Fractal dimension of polymer matrix
ΔD	Degree of multifractality
MSD	Mean square displacement
ρ	Amount of polymer matrix
σ	Width of the Cauchy distribution/standard deviation of the Gaussian distribution
